# Dietary Knowledge, Attitude, Practice Survey and Nutritional Knowledge-Based Intervention: A Cross-Sectional and Randomized Controlled Trial Study among College Undergraduates in China

**DOI:** 10.3390/nu16142365

**Published:** 2024-07-21

**Authors:** Junjie Wu, Wei Yu, Zhuo Xu, Yuxuan Chen, Jiaomei Li, Qinghua Sun

**Affiliations:** 1 School of Public Health, Zhejiang Chinese Medical University, Hangzhou 310053, China; 2 The Fourth School of Clinical Medicine, Zhejiang Chinese Medical University, Hangzhou 310053, China; weiweichaoku@126.com

**Keywords:** KAP, dietary knowledge, dietary attitude, dietary practice, health literacy, undergraduates

## Abstract

Background: Understanding undergraduates’ dietary literacy, including dietary knowledge, attitude and practice (KAP), is important for future health promotion policies. Therefore, this study aimed to investigate the current status and influencing factors of dietary literacy in Chinese college undergraduates and explore whether a nutritional lecture could improve their dietary literacy. Methods: This study included two parts: a cross-sectional study (*n* = 1026) conducted by a dietary literacy questionnaire, and a randomized controlled trail (RCT) that enrolled 99 college undergraduates who were randomized to a control group or a nutritional lecture group. Data from the questionnaire and 72 h food records were obtained on day 0, day 3 and day 100 before and after intervention. Results: This cross-sectional study showed that the mean scores of dietary KAP were respectively 100.0 (33.3), 59.1 (13.6) and 71.7 (11.7), with an excellent rate of 36.6%, 1.9% and 3.4%. Female upper-grade undergraduates and those with medicine-related majors illustrated higher dietary knowledge scores (*p* < 0.001). Dietary attitude rather than dietary knowledge had a significant impact on dietary practice (*p* < 0.001). This finding was in line with the results in the RCT study. Compared with the control group, dietary knowledge was significantly improved in the nutritional lecture group on both day 3 (*p* = 0.002) and day 100 (*p* = 0.023) after intervention. However, dietary behavior was improved only on day 3 post nutritional lecture (*p* = 0.029) but decreased to the original level 100 days later (*p* < 0.001). Conclusions: This study discovered the unsatisfying status and discrepancy between dietary literacy among college undergraduates of different sex, majors and college years. Dietary attitude instead of dietary knowledge was discovered as a determining role in dietary practice. One nutritional lecture could improve undergraduates’ dietary literacy but the effect was not long-lasting. Further studies with more reinforced and durable interventions are warranted.

## 1. Introduction

A healthy diet habit is one of the essentials to a person’s physical and mental health, along with aging [1]. The World Health Organization (WHO) believes that a healthy diet for adults should be rich in fruits, vegetables, beans, nuts and whole grains [2]. Many previous studies have found that a healthy diet, such as the Mediterranean diet, can help to prevent obesity [3], regulate gut microbiota [4] and reduce the incidence of cancer [5], chronic kidney diseases [6] and many other diseases. Conversely, unhealthy diets and eating habits may increase the risk of obesity, cardiovascular disease, lung cancer and metabolic syndrome [7]. In addition to physical health, many studies confirmed the correlation between healthy diet and mental health in adolescents and children [8].

In the past decades, China’s economy has developed substantially. Along with these changes, the Chinese diet is also gradually changing from a plant-based to a meat-based diet [9]. At the same time, the mortality and morbidity of many chronic diseases, such as cardiovascular diseases and diabetes, have dramatically increased and have become more prevalent in the young- and middle-aged groups [10]. As an essential component in young people’s daily life, a healthy diet represents, and even likely leads, the formation of a healthy lifestyle in young people [11]. However, contemporary young people, especially undergraduates, are facing challenges brought about by new lifestyles, which makes them more likely to develop bad dietary habits [12]. Previous studies have shown that the dietary situation of global adolescents is problematic [13]. Many studies have also suggested that the dietary status of college undergraduates is problematic [14]. Therefore, more attention should be paid to undergraduates’ dietary status.

The KAP model, which focuses on health literacy integrating knowledge, attitude and practice, points out that knowledge is the foundation for the establishment of positive and correct attitudes, and attitudes are the driving forces for behavior change [15]. In a former study, health literacy was found to have a significant correlation with people’s health behaviors and the incidences of many diseases [16,17].

Dietary literacy, as a special health literacy, is defined as the capacity to obtain, process and understand nutrition information and the materials needed to make appropriate decisions regarding one’s health [18], which is strongly related to one’s eating behaviors [19,20]. College students tend to behave better in the ability of appraising and applying health information compared with those with less education [21]. However, the dietary literacy status of undergraduates has been shown to remain unsatisfactory by many previous studies, which makes current college students a population of interest [22,23]. And in order to solve this problem, a lot of studies have studied and found out several measures, among which nutrition education, in particular, was discovered as an effective way to improve college students’ dietary literacy [24,25].

Located in the southeast of China, Zhejiang province has a high level of modernization, with numerous colleges and a large undergraduate population. In the previous study, Zhejiang was shown to have a high incidence rate of overall nutritional deficiency [26], indicating the unsatisfactory dietary status of college undergraduates. However, few researches were carried out to investigate the Chinese undergraduates’ dietary literacy status, especially in Zhejiang, from the perspective of KAP.

Therefore, this study aimed to investigate the current status and the influencing factors of Chinese undergraduates’ dietary literacy, especially in Zhejiang, through a cross-sectional study and explore whether a nutritional lecture could improve their dietary literacy via a randomized controlled trial (RCT). We hypothesized that undergraduates’ dietary literacy was poor and influenced by multiple factors, which might be changed by the intervention of a targeted nutrition knowledge lecture. We hoped this study could have some implication for a positive and long-term significance for future health promotion policies.

## 2. Material and Method

### 2.1. Study Design

**Figure 1:**
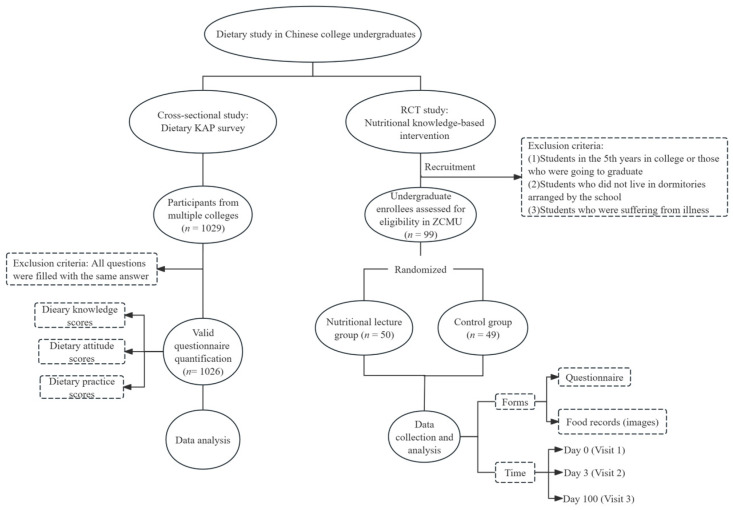
Study design. Notes: KAP (knowledge, attitude, practice); ZCMU (Zhejiang Chinese Medical University).

This study was designed in two paths (as seen in Figure 1) as a cross-sectional study and a randomized controlled trial (RCT), which were reviewed and approved by the Medical Ethics Committee of Zhejiang Chinese Medical University, 20221011-3, 11 October 2022 and are registered with the clinical trials registry (http://clinicaltrials.gov, ID: NCT05791500, accessed on 30 March 2023).

### 2.2. A Cross-Sectional Study Conducted by Questionnaire on Dietary Literacy in College Students

Before the formal investigation, twenty-two college students were recruited from a few universities in China, including Zhejiang Chinese Medical University (ZCMU), Zhejiang University, Wenzhou Medical University, Zhejiang University of Technology and Ningbo University, to fill out a preliminary “Undergraduate dietary literacy KAP questionnaire” as an initial survey. According to the results, only 17.4% of college students had a relatively healthy diet (divided by 80% of the dietary literacy score). The required sample size was calculated to be 959, based on a two-sided *z*-test with a significance level of 5% and an allowable error of 0.024. 

Based on the global school-based student health survey (GSHS) questionnaire [27], an “Undergraduate dietary literacy KAP questionnaire” for data collection was designed, which contained a total of 35 questions and was divided into 4 parts: basic demographic characteristic questions of the respondents like sex, college year and major; dietary knowledge questions; dietary attitude questions and dietary practice questions. The detailed questionnaire can be seen in Supplemental Table S1.

Then, college undergraduates from multiple universities in Zhejiang were enrolled and asked to fill out the “Undergraduate dietary literacy KAP questionnaire” via the platform “Questionnaire Star” of WeChat https://www.wjx.cn/vm/YDgDLEJ.aspx# (accessed on 10 May 2022). After invalid questionnaire respondents (all options were filled with the same answer) were excluded, remaining valid questionnaires were scored to obtain dietary knowledge, attitude and practice scores according to the criteria in Supplemental Table S1. To have a clear understanding of the current dietary KAP status among undergraduates, all the scores were standardized by the hundred-mark system and participants were ranked as “excellent”, “good”, “average” and “poor” based on 90 points, 80 points and 60 points. 

### 2.3. An RCT Study Performed by a Nutritional Lecture 

In the RCT study, the sample size was calculated to be 90. The calculation was conducted with a probability of 50% and 17.4%, respectively, for college students in the nutritional lecture group and the control group owning healthy dietary KAP, based on type I error type (*α*) of 0.05 and type II error (*β*) of 0.1.

Participants were recruited through a poster on the campus at ZCMU. Those undergraduates who were unable to complete the whole study (students in the 5th year in college or those who were going to graduate; students who did not live in dormitories arranged by the school; students who were suffering from illness) were excluded. All the participants were adult undergraduates and they signed the written consent. After that, participants were randomized to the nutritional lecture group and the control group via a random number generated by Excel 2019. Odd-numbered enrollees were assigned to the nutritional lecture group and even-numbered ones were assigned to the control group.

Participants in the nutritional lecture group were requested to attend one single nutritional knowledge-based lecture, which was provided by a faculty from the Division of the Nutrition and Food Hygiene in the School of Public Health at ZCMU. The specific topics of the lecture mainly included knowledge on components of common dietary foods in daily life, how to choose healthy foods and the relationship between unhealthy diet and multiple chronic metabolic disease.

Baseline data (day 0) and data after intervention (day 3 and day 100) were collected in two forms: “Undergraduate dietary literacy KAP questionnaire” and 72 h food records through food images. The scores of dietary knowledge and attitude were calculated from questionnaires in the same way as indicated above. The dietary behaviors of participants were evaluated more scientifically and accurately using two methods. One was assessed by dietary practice scores via the “Undergraduate dietary literacy KAP questionnaire”. The other was to collect food images of the 72 h dietary records from the participants, which were estimated by the Dietary Quality Index-International (DQI-I) method to obtain a dietary quality score [28]. The detailed scoring rules for each item are shown in Supplementary Table S2. 

### 2.4. Statisitcal Analysis

Categorical variables are presented as a sample percentage (%), and continuous variables are presented as means with standard deviation (SD) or medians with interquartile range (IQR) for the variables with normal or non-normal distribution, respectively. The normality of variable distribution was verified with the Shapiro–Wilk test before the statistical analysis. Then, *t*-tests or Wilcoxon tests were used to analyze the differences between the two groups. ANOVA analysis and Kruskal–Wallis tests were used to analyze the differences among multiple groups. Spearman rank correlation analysis and multivariate linear regression were applied to identify the relationship among dietary knowledge, attitude, practice and factors that affect dietary literacy. All the statistical methods in this study were two-tailed tests with a significance of 0.05 using SPSS 25.0. and GraphPad Prism 9.

## 3. Results

### 3.1. Basic Characteristics of Questionnaire Respondents 

After excluding three invalid questionnaires, a total of 1026 questionnaires were deemed valid. Among the responses, 944 were from ZCMU and 82 were from other universities nationwide. The numbers of male and female respondents were 271 and 755. The numbers of college undergraduates in their first, second, third, fourth and fifth year were 227, 511, 100, 144 and 44, respectively. The numbers of undergraduates majoring in the literature and arts, science and engineering, and medicine-related majors were 44, 78 and 904, respectively. The detailed results are shown in Table 1.

### 3.2. Current Dietary KAP Status of Questionnaire Respondents

**Figure 2:**
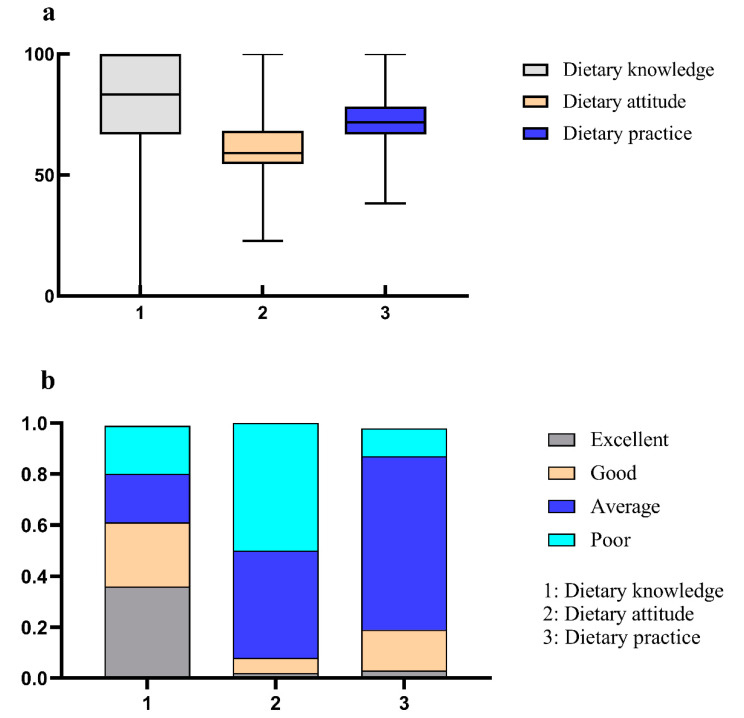
Distribution of dietary KAP literacy scores among college students. (**a**) Boxplots of questionnaire respondents’ dietary knowledge, attitude and practice scores. (**b**) Percentage bar graphs of undergraduates with different levels of dietary KAP.

The dietary knowledge, attitude and practice of questionnaire participants were respectively 100.0 (33.3), 59.1 (13.6) and 71.7 (11.7). The rates of undergraduates with “excellent” dietary knowledge, attitude and practice were, respectively, 36.6%, 1.9% and 3.4%. The detailed results are shown in Figure 2.

### 3.3. Dietary Literacy Discrepancy among Undergraduates of Different Sex, College Years and Majors

**Figure 3:**
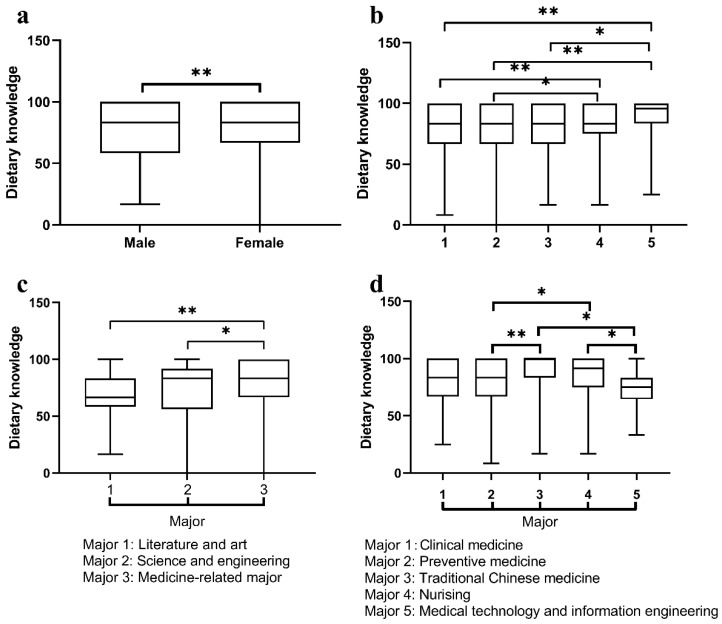
Comparisons of dietary knowledge scores of undergraduates among different sex, college years and majors. (**a**) Sex and dietary knowledge scores: female undergraduates achieved higher dietary knowledge scores than male students. (**b**) College year and dietary knowledge scores: upper-grade undergraduates achieved higher dietary knowledge scores than lower-grade students. (**c**) Major and dietary knowledge scores: medicine-related undergraduates achieved higher dietary knowledge scores than students majoring in literature and art and science and engineering. (**d**) Different medicine-related major and dietary knowledge scores: undergraduates majoring in nursing and traditional Chinese medicine achieved higher dietary knowledge grades than students majoring in preventive medicine and medical technology and information engineering. * *p* < 0.05, ** *p* < 0.001.

We grouped and analyzed the differences in dietary knowledge, attitude and practice scores based on sex, college years and majors. First, the dietary knowledge scores of female and male participants were, respectively, 83.3 (33.3) and 83.3 (41.6). Female undergraduates showed better dietary knowledge scores than male counterparts (*p* < 0.001). Second, dietary knowledge scores among the college undergraduates in the first, second, third, fourth and fifth year were 83.3 (41.7), 83.3 (33.3), 83.3 (33.3), 83.3 (25.0) and 95.8 (16.7), respectively. It was found that senior students behaved better than junior students in dietary knowledge (*p* < 0.001). Additionally, students majoring in medicine had a higher level of dietary knowledge than students of non-medicine-related majors (*p* < 0.001). Dietary knowledge scores among the majors of literature and art, science and engineering, and medicine-related majors were 66.7 (25.0), 83.3 (35.4) and 83.3 (33.3). As for different medicine-related majors, traditional Chinese medicine (TCM) students had a better grasp of dietary knowledge than preventive medicine students (*p* < 0.001) and medical technology and information engineering (MTIE) students (*p* < 0.05). Similarly, students majoring in nursing achieved higher dietary knowledge scores than preventive medicine and MTIE students (*p* < 0.05). The detailed results are seen in Figure 3. However, there were no significant differences in dietary attitude and practice scores among the students of different sex, college years and majors. The specific results are shown in Supplementary Tables S3–S6.

### 3.4. The Relationship between Dietary Knowledge, Attitude and Practice 

**Figure 4:**
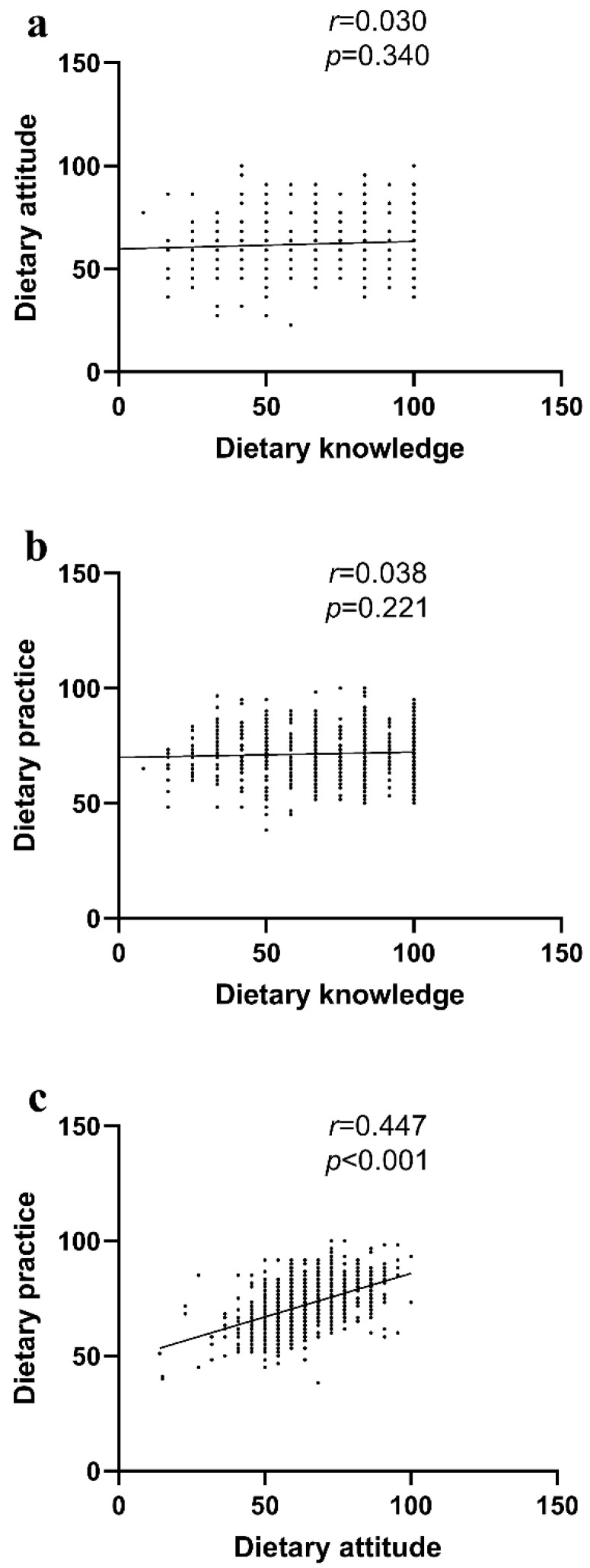
Scatter plot of the association among dietary KAP scores. (**a**) Insignificant association between dietary knowledge and dietary attitude. (**b**) Insignificant association between dietary knowledge and dietary practice. (**c**) Significant association between dietary attitude and dietary practice.

As shown in the scatter plot in Figure 4, there was a positive correlation between dietary attitude and dietary practice. Spearman rank correlation analysis also indicated a significant correlation between dietary practice and dietary attitude (r = 0.447, *p* < 0.001), while there was no significant correlation between dietary practice and dietary knowledge or between dietary knowledge and dietary attitude. Multiple linear regression analysis, including factors of college year, sex, major, dietary knowledge and attitude, showed that dietary attitudes had a significant impact on dietary practice (*β* = 0.999, *t* = 15.092, *p* < 0.001), while dietary knowledge, different college years, majors and sex did not. The detailed results are shown in Table 2.

### 3.5. Basic Characteristics of the Participants in the RCT Study

Ninety-nine undergraduate students from ZCMU were finally recruited in this RCT study and were randomized into the nutritional lecture group of fifty students or the group control of forty-nine students. Characteristics, including participants’ sex, years in colleges and major, were collected, and there was no significant difference between the two groups in terms of their basic characteristics, which are shown in Table 3.

### 3.6. The Impact of the Nutritional Lecture Shown in the Questionnaire

**Figure 5:**
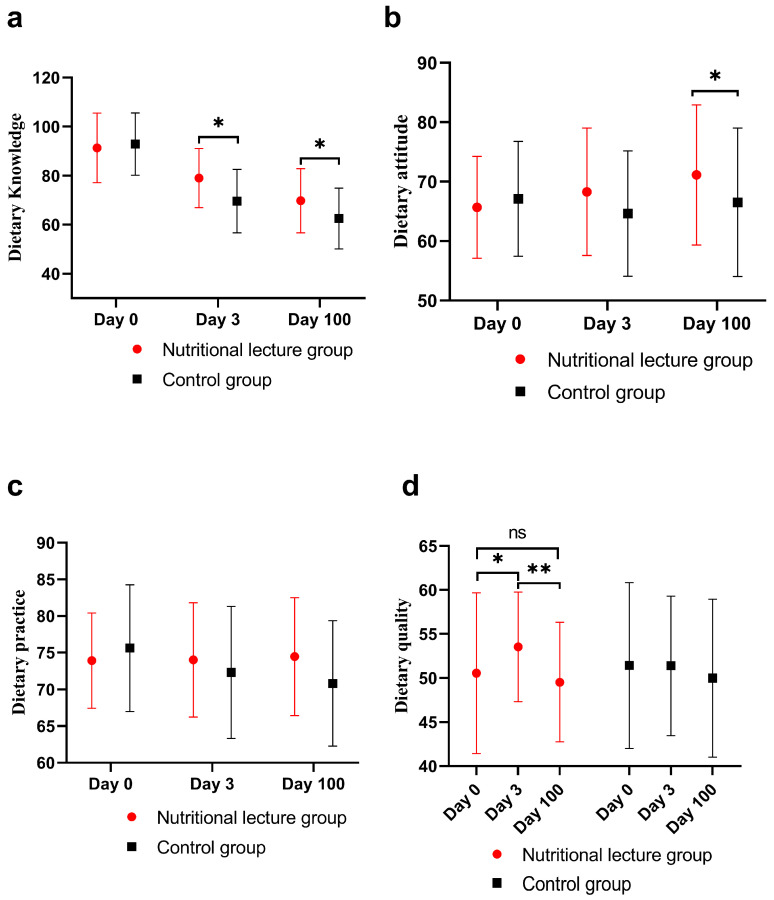
Impact of nutritional lecture on dietary literacy. (**a**) Significant difference in dietary knowledge scores shown on day 3 and day 100 after intervention and insignificant difference shown on day 0 between the two groups. (**b**) Significant difference in dietary attitude scores shown on day 100 and insignificant difference on day 0 and day 3 between the two groups. (**c**) Insignificant difference in the dietary practice scores between the two groups shown on days 0, 3 and 100. (**d**) Trends of dietary quality of the two groups. The nutritional lecture group showed a significant improvement in dietary quality scores on day 3 and a significant decrease on day 100. * *p* < 0.05, ** *p* < 0.001, ns (not significant).

As shown in Figure 5, before intervention, there was no significant difference between the two groups in the baseline data of dietary knowledge, attitude and practice scores. After intervention, participants in the nutritional lecture group illustrated better dietary knowledge scores than those in the control group on day 3 (*p* = 0.002) and day 100 (*p* = 0.023); additionally, a significantly higher level of dietary attitude was shown in the intervention group on day 100 (*p* = 0.044), but on day 3 the difference was not significant (*p* = 0.058). And there was also no significant difference in dietary practice scores on day 3 (*p* = 0.367) and day 100 (*p* = 0.052). The detailed results are shown in Supplementary Table S7.

### 3.7. Improvement Shown in the Food Images

ANOVA analysis conducted on the dietary quality scores in the three visits revealed that nutritional lecture group participants showed a significant improvement on day 3 compared with the baseline (difference of 3.0, 95% CI of 0.3 to 5.7, *p* = 0.029). However, there was a significant decrease on day 100 compared with day 3 (difference of −4.0, 95% CI of −2.0 to −6.0, *p* < 0.001) and the improvement on day 3 seemed to have disappeared. The results are shown in Figure 5. However, there was no statistical difference in the dietary quality scores between the two groups on day 0, day 3 and day 100. The detailed results are shown in Supplementary Tables S8 and S9.

## 4. Discussion

There were several findings in our present study. First, in the cross-sectional study, we could see the problematic situation of dietary literacy in college students from the low percents of undergraduates with “excellent” and “good” dietary attitude and practice scores. Additionally, significant differences were discovered in dietary knowledge among the undergraduates in terms of different sex, college years and majors. Moreover, we revealed the determining role of dietary attitude on undergraduates’ dietary practice. Furthermore, in the RCT trial, dietary knowledge, attitude and dietary behaviors of the participants were significantly improved in a short period of time after a nutritional lecture. However, their dietary behaviors returned to the original state over several months after intervention.

The college undergraduates’ dietary literacy situation seemed to be unsatisfactory. Many college students were found to hold a neutral attitude towards their eating health and attached little importance to the nutritional value of foods. Similar results have been reported in a previous study among adolescents in Chongqing, China [29]. Additionally, many unhealthy eating behaviors such as skipping breakfasts were found among undergraduates, which is consistent with previous studies [30]. In this study, undergraduates seemed to behave well in terms of dietary knowledge. But it did not mean Chinese college students had a good command of nutritional knowledge, because the basic and limited number of dietary knowledge questions might have been too easy for questionnaire respondents, especially students of medicine-related majors who were exposed to courses related to nutrition.

The discrepancies in dietary knowledge among the undergraduates of different sex, college years and majors are of great interest to be explored. As to our finding that the female students showed better dietary knowledge than their male counterparts, it might be related to the fact that Chinese female undergraduates performed better than male undergraduates in learning psychology [31]. Additionally, in order to keep fit, women seemed to pay more attention to contents related to healthy diets [30]. Moreover, regarding the difference in dietary knowledge between the senior and junior students, one reason could be that nutrition education was deemed an important part for college students’ studying, especially for medical students [32], and that those junior undergraduates had not yet enrolled in the courses related to health promotion and nutrition while the senior undergraduates had already finished those courses. In former studies [33,34], these curricula were discovered to effectively improve students’ knowledge. Another reason could be that the senior students might be better educated and more knowledgeable compared with the junior ones after consecutive years of studying, thus leading to their higher dietary knowledge scores in the survey. Similar to the difference between upper-grade and lower-grade students, a couple of studies conducted in China reported the same difference in dietary knowledge between adults with a higher education level such as the level of doctor and those with a lower education level like the level of senior high school [35,36]. They pointed out that education level might contribute to adults’ stronger interests in dietary knowledge and better understanding of nutritional requirements. The students of medicine-related majors illustrated a higher dietary knowledge level, consistent with a former study that explained that medical students might be more conscious of healthy eating, and they might also find it simpler to search for, find and comprehend information related to wellbeing [23]. It might also be related to the fact that medical students were required to take courses related to nutrition in their programs [32], and they were more likely to be exposed to an environment that was filled with knowledge on healthy diets even in their out-of-classroom activities than those of non-medicine-related majors, which might eventually lead to the difference of dietary knowledge between them. One of the interesting results we saw was that the students majoring in TCM showed better dietary knowledge scores than the others, including preventive medicine, which was a surprise to us. To explore the causes, we found that TCM majors compared with other majors were very competitive, and had higher college admission scores in the college entrance examination than the others. They might be better educated and have a better learning ability, making them more capable in appraising and grasping health information compared with those with less education [21]. In addition, the curricula of TCM are very intense, which may promote the students to study harder and more diligently. 

Despite the difference in dietary knowledge, in our study, there was no significant difference in dietary attitude scores among the undergraduate students regarding their sex, college year or major. This might suggest that the students who are female, senior or major in medicine-related programs, although having a deeper understanding and acquisition of dietary knowledge and being able to recognize healthy diets in their daily lives, might not realize the importance and necessity of healthy diets. They might think that, at their current and young age, they do not need to pay much attention to healthy eating, which ultimately leads to an insignificant difference in dietary attitude scores [37].

Consequently, there was also no significant difference in dietary practice scores among the college students of different sex, college years and majors. Due to the lesser importance attached to healthy eating, they seem to prefer some delicious “junk food” over healthy foods that might have poor taste and flavor, which ultimately leads to insignificant differences in their dietary practice scores [37]. 

The results of multiple linear regression shown in Table 2 also explain the phenomenon of no differences in dietary attitude and practice between undergraduates with better and poorer dietary knowledge. It was shown that the students’ attitudes towards healthy diets are the determining factor that affects dietary behavior, while dietary knowledge is just the basic or secondary factor for the occurrence of dietary practice, which is consistent with the KAP health education model [15].

We deeply recognize the importance of knowledge. However, we also realize that there may be a “gap” between knowledge or belief and behavior or action. Therefore, an intervention via a short-term targeted nutritional knowledge-based lecture was designed and aimed to illustrate how dietary knowledge improvement interacts with undergraduates’ dietary attitude and practice. From the results, we could see that one nutritional lecture might be an effective way to promote the occurrence of healthy dietary behaviors among undergraduate students in a short period of time by improving the participants’ knowledge, which seemed to be consistent with many previous studies [38,39,40]. However, the effect was not long-lasting, which implies that, in order to maintain improved dietary literacy, more targeted and reinforced education is needed. Therefore, we explored several factors that might be required by effective nutrition education. Firstly, studies with longer durations seemed to achieve their started objectives better [41,42]. One short nutritional lecture was probably not enough to improve undergraduates’ dietary attitudes and help them to establish a healthy dietary behavior pattern. Their dietary behaviors were improved in a short time, but not long after, they would return to their original status. Additionally, there was a lack of interaction between the investigators and participants, which might have led to the decrease in the effectiveness of a nutritional lecture [43]. It was also possible that, due to the large time span between day 3 and day 100, there were many confounding factors; for example, it was the time period for students in ZCMU to return to school for exams on day 100. They had just passed a winter vacation and had not yet adapted to the differences in diet between home and school, with a decrease in attention to healthy eating under the pressure of final exams. And stress might result in irregular and unhealthy eating behaviors [44]. For example, they might be more inclined to choose “junk food” with better taste and flavor or might overeat or even not eat.

In the RCT study, the significant differences of dietary knowledge scores indicated the fact that the nutritional lecture truly improved the dietary knowledge of the participants in the nutritional lecture group. However, there was only a marginally significant difference in dietary attitude scores on day 3 between the two groups and, on day 100, there was a significant difference. This strange phenomenon could be explained by the Hawthorne effect [45]. After just joining this study, participants might have thought that they were being observed by others and thus developed a tendency to change their behavior even without any interventions like a nutritional lecture in this study, ultimately resulting in insignificant differences between the nutritional lecture group and the control group. But, after a few months, participants in the control group returned to their original state, while those in the nutritional lecture group still maintained a better attitude towards healthy diets due to the influence of nutritional lectures, thereby leading to a difference between the two groups. Additionally, the *p*-value of the dietary attitudes between the two groups on day 3 was 0.056. Although it did not reach a significant level, it was very close. If the sample size of the study population is increased or the frequency of the nutritional lecture is increased in subsequent studies, it will be possible to obtain a positive result. Similarly, regarding the dietary practice scores shown in the questionnaires, there was also a marginally significant difference between the two groups on day 100. This might also have been due to the Hawthorne effect and the limited sample size of participants in this study.

In this study, we combined the KAP health education model to investigate the dietary status of Chinese undergraduates, especially in Zhejiang province, from multiple perspectives of dietary knowledge, dietary attitude and dietary practice, and studied the relationship among the three factors along with many other underlying factors that might influence dietary literacy, which seemed not very clear in previous studies. Moreover, in addition to a cross-sectional survey, preliminary targeted nutritional knowledge-based interventions were carried out on participants recruited from ZCMU in this study to explore whether a nutritional knowledge-based lecture could improve dietary literacy. Additionally, in the RCT study, various methods, including questionnaires and dietary records based on food images, were used to estimate participants’ dietary behaviors more scientifically and accurately. After that, the intervention impact was evaluated, providing reference significance for more long-term and effective intervention measures in the future. 

However, there were still some limitations in our study. First, in the cross-sectional study, we adopted the method of convenience sampling when collecting the questionnaires. The “Undergraduate dietary literacy KAP questionnaire” was mainly distributed in universities in Zhejiang, with ZCMU as the main focus, and the proportion of women in both the questionnaire and the intervention participants was higher than men. These factors might have caused selection bias. Furthermore, the data after intervention were collected only twice, which could not illustrate clearly or well the trend of changes in undergraduates’ dietary literacy in both the intervention and the control groups. Lastly, the intervention measure adopted in this study was only one nutritional lecture. The longer term and far-reaching impact of nutrition education might not have been revealed.

## 5. Conclusions

In summary, this study revealed the unsatisfactory current dietary literacy status among Chinese undergraduate students, especially in Zhejiang, and demonstrated some discrepancies in dietary knowledge in college students in terms of sex, college year and major, discovering the determining role of dietary attitude on dietary practice. One targeted nutritional knowledge-based lecture was only effective short term regarding the improvement of their dietary literacy. In order to achieve substantial and long-lasting improvements in the healthy dietary behavior in college students in China, additional efforts are still needed, such as offering lasting rather than short-term nutrition courses among college students, strengthening the interaction between students and investigators during the process of education and setting nutrition labels in college canteens to help students establish a clearer understanding of what they eat every day.

## Figures and Tables

**Table 1:** Basic demographic characteristics of questionnaire respondents.

Variable	M (IQR)	Quantity	Percent (%)
Undergraduates of Zhejiang Chinese Medical University			
Yes		944	92.0
No		82	8.0
Sex			
Male		271	26.5
Female		755	73.5
College year			
Freshman		227	22.2
Sophomore		511	49.8
Junior		100	9.7
Senior		144	14.0
5th year		44	4.3
College major			
Literature and art		44	4.3
Science and engineering		78	7.6
Basic medicine		11	1.1
Clinical medicine		154	15.0
Preventive medicine		425	41.4
Stomatology		5	0.5
Traditional Chinese medicine		122	11.9
Medical technology		22	2.1
Nursing		109	10.7
Other medicine-related majors		56	5.5
Dietary knowledge	83.3 (33.3)		
Dietary attitude	59.1 (13.6)		
Dietary practice	71.7 (11.7)		

Notes: M (median); IQR (interquartile range).

**Table 2:** Multivariate linear regression analysis of the influencing factors of dietary practice.

Factor	*β*	SE	Adjusted *β*	*t*	*p*
Constant	71.818	1.385	/	51.836	<0.001
Dietary knowledge	0.015	0.012	0.035	1.220	0.223
Dietary attitude	0.366	0.022	0.457	16.353	<0.001
Sex					
1 = Male 2 = Female	/				
	−0.623	0.585	−0.030	−1.065	0.287
Major					
1 = Literature and art	/				
2 = Science and engineering	0.210	1.544	0.006	0.136	0.892
3 = Medicine-related major	−0.056	1.271	−0.002	−0.044	0.965
College year *	−0.338	0.238	−0.040	−1.420	0.156

Notes: R^2^ = 0.214, adjusted R^2^ = 0.210, F = 46.302, *p* < 0.001. *: 1 = freshman, 2 = sophomore, 3 = junior, 4 = senior, 5 = 5th year.

**Table 3:** Demographic characteristic analysis of the nutritional lecture group and the control group participants.

Variable	Group (*n* (%))		*p*
	Intervention Group	Control Group	
Sex			
Male	10 (20.0%)	8 (16.3%)	0.636
Female	40 (80.0%)	41 (83.7%)	
Year			
Freshman	16 (32.0%)	11 (22.4%)	0.772
Sophomore	15 (30.0%)	17 (34.7%)	
Junior	17 (34.0%)	19 (38.8%)	
Senior	2 (4.0%)	2 (4.1%)	
Major			
Nursing	1 (2.0%)	4 (8.2%)	0.345
Computer science and technology	1 (2.0%)	0 (0.0%)	
Stomatology	2 (4.0%)	1 (2.0%)	
Clinical medicine	5 (10.0%)	8 (16.3%)	
Biotechnology	1 (2.0%)	2 (4.1%)	
Life science	2 (4.0%)	6 (12.2%)	
Hearing and speech rehabilitation	1 (2.0%)	1 (2.0%)	
Health inspection and quarantine	2 (4.0%)	0 (0.0%)	
Medical laboratory technology	1 (2.0%)	1 (2.0%)	
Medical imaging	0 (0.0%)	1 (2.0%)	
Preventive medicine	23 (46.0%)	15 (30.6%)	
Acupuncture and massage	1 (2.0%)	2 (4.1%)	
Integrated Chinese and Westen clinical medicine	3 (6.0%)	0 (0.0%)	
TCM	6 (12.0%)	5 (10.2%)	
Midwifery	1 (2.0%)	3 (6.1%)	

Notes: TCM (traditional Chinese medicine).

## Data Availability

The datasets used in the present study are available from the corresponding author on reasonable request.

## Supplementary Materials

The following supporting information can be downloaded at: https://www.mdpi.com/article/10.3390/nu16142365/s1, Supplementary Table S1: Scoring criteria of “Undergraduate dietary literacy KAP questionnaire”; Supplementary Table S2: Components of Diet Quality Index-International (DQI-I); Supplementary Table S3: Sex and dietary knowledge, attitude and practice scores; Supplementary Table S4: Student college year and dietary knowledge, attitude and practice scores; Supplementary Table S5: Student college major and dietary knowledge, attitude and practice scores; Supplementary Table S6: Different medicine-related major and dietary knowledge, attitude and practice scores; Supplementary Table S7: Dietary knowledge, attitude and practice scores of the participants; Supplementary Table S8: Intervention on participants’ dietary quality scores; Supplementary Table S9: Dietary quality score comparison.
