# Supplementary material for: Dietary Knowledge, Attitude, Practice Survey and Nutritional Knowledge-Based Intervention: A Cross-Sectional and Randomized Controlled Trial Study among College Undergraduates in China
Source: Nutrients. 2024 Jul 21;16(14):2365. doi: 10.3390/nu16142365 (PMC11279395; doi:10.3390/nu16142365)
Supplement: Supplementary file 1 [file nutrients-16-02365-s001.zip › nutrients-3105155-supplementary.pdf]

Supplementary Table S1. Scoring criteria of “Undergraduate dietary literacy KAP questionnaire”.

Question	Score	Scoring criteria
Q1-Q10 (Demographic questions)	/	/
**Dietary practice standardized**	**1-100**	dietary practice score *100/60
**Dietary practice**	**1–60**	/
Q11 (question of healthy food (vegetables, fruit and high fiber cereals) frequency)	1–6	Twice or more than twice a day = 6 points
		Once a day = 5 points
		4 to 6 times a week = 4 points
		2 to 3 times a week = 3 points
		Once a week= 2 points
		Less than once a week = 1 point
Q12 (question of carbonated beverages or bubble tea frequency)	1–6	Less than once a week = 6 points
		Once a week = 5 points
		2 to 3 times a week = 4 points
		4 to 6 times a week = 3 points
		Once a week = 2 points
		Twice or more than twice a day = 1 point
Q13 (question of consumption of three meals)	1–4	Eat regularly every day = 4 points
		4 to 6 times a week = 3 points
		1 to 3 times a week = 2 points
		Never eat = 1 point
Q14 (question of frequency of eating late night snacks, replacing meals with convenient food and ordering takeout)	1-4	0 times a week = 4 points
		1 to 2 times a week = 3 points
		3 to 5 times a week = 2 points
		6 or more times a week = 1 point
Q15 (question of volume of water or tea consumed daily)	1-4	More than 1500ml = 4 points
		1000 to 1500ml = 3 points
		500 to 1000ml = 2 points
		0 to 500ml =1 point
Q16 (question of choosing the food combination which is closest to their usual breakfasts)	1-4	The healthiest food combination = 4 points
		The second healthiest food combination = 3 points
		The second unhealthiest food combination = 2 points
		The unhealthiest food combination =1 point
Q17 (question of choosing the food combination	1-4	The healthiest food combination = 4 points

Question	Score	Scoring criteria
which is closest to their usual dinners)		The second healthiest food combination = 3 points
		The second unhealthiest food combination = 2 points
		The unhealthiest food combination =1 point
**Dietary attitude standardized**	**0-100**	dietary attitude score *100/18
**Dietary attitude**	**0-18**	/
Q19 (question of the biggest influencing factor when choosing main meal food)	0-2	The health benefit of food = 2 points
		The calories of food = 1 point
		The flavor or the price of food = 0 points
Q20 (question of difficulty of forming healthy eating habits)	1-4	Definitely easy = 4 points
		Rather easy = 3 points
		Rather difficult = 2 points
		Definitely difficult = 1 point
Q21 (question of the necessity of forming healthy eating habits)	1-4	Definitely necessary = 4 points
		Rather necessary = 3 points
		Rather unnecessary = 2 points
		Definitely unnecessary= 1 point
Q22 (question of the attention paid to healthy eating)	1-4	Always pay attention to = 4 points
		Occasionally pay attention to = 3 points
		Only in a bad health attention = 2 points
		Never pay attention to = 1 point
Q25 (question of the response when hearing the harm of their favorite food)	1-4	Strictly forbidden = 4 points
		Try not to eat = 3 points
		Approximately restrain = 2 points
		Keep eating as usual = 1 point
**Dietary knowledge standardized**	**0-100**	dietary knowledge score *100/14
**Dietary knowledge**	**0-14**	/
Q26 to Q28 (nutritional knowledge questions)	0-2	Correct answer = 2 points
		Incorrect answer = 0 points
Q29 to Q30 (questions of choosing the healthiest food combination)	1-4	Heathiest one =3 points
		The second healthiest one =2 points

Question	Score	Scoring criteria
		The second unhealthiest one = 1 point
		The unhealthiest one = 0 points
**Dietary literacy standardized**	**1-100**	dietary literacy score *100/92
**Dietary literacy**	**1-92**	dietary knowledge score + dietary attitude score + dietary practice score

Supplementary Table S2. Components of Diet Quality Index-International (DQI-I).

**Component**	**Score**	**Scoring criteria**
**Variety**	**0-20**	/
Overall food group (A)	0-15	≥1 serving from each food group/d = 15
		Any 1 food group missing/d = 12
		Any 2 food groups missing/d = 9
		Any 3 food groups missing/d = 6
		≥4 food groups missing/d = 3
		None from any food groups = 0
Within-group for protein source (B)	0-5	≥3 different sources/d = 5
		2 different sources/d = 3
		From 1 source/d = 1
		None = 0
**Adequacy**	**0-40**	/
Vegetables	0-5	≥3-5 servings/d = 5, 0 servings/d = 0
		≥100%
		<100-50%
		<50%
Fruits	0-5	≥2-4 servings/d = 5, 0 servings/d = 0
		≥100%
		<100-50%
		<50%
Grains	0-5	≥6-11 servings/d = 5, 0 servings/d = 0
		≥100%
		<100-50%
		<50%
Fibers	0-5	≥20-30 g/d = 5, 0 g/d = 0
		≥100%
		<100-50%
		<50%
Proteins	0-5	≥10% of energy/d = 5, 0% of energy/d = 0
		≥100%
		<100-50%
		<50%
Iron	0-5	≥100% RDA (AI)/d = 5, 0% RDA (AI)/d = 0

Component	Score	Scoring criteria
		≥100%
		<100-50%
		<50%
		≥100% AI/d = 5, 0% AI/d = 0
Calcium	0-5	≥100%
		<100-50%
		<50%
		≥100% RDA (RNI)/d = 5, 0% RDA (RNI)/d = 0
Vitamin C	0-5	≥100%
		<100-50%
		<50%
**Moderation**	**0-30**	/
		≤20% of total energy/d = 6
Total fat	0-6	>20-30% of total energy/d = 3
		>30% of total energy/d = 0
		≤7% of total energy/d = 6
Saturated fat	0-6	>7-10% of total energy/d = 3
		>10% of total energy/d = 0
		≤300 mg/d = 6
Cholesterol	0-6	>300-400 mg/d = 3
		>400 mg/d = 0
		≤2400 mg/d = 6
Sodium	0-6	>2400-3400 mg/d = 3
		>3400 mg/d = 0
		≤3% of total energy/d = 6
Empty calorie foods	0-6	>3-10% of total energy/d = 3
		>10% of total energy/d = 0
**Overall balance**	**0-10**	/
		55-65:10-15:15-25 = 6
		52-68:9-16:13-27 = 4
Macronutrient ratio (C)	0-6	50-70:8-17:12-30 = 2
		Otherwise = 0
Fatty acid ratio (D)	0-4	P/S = 1-1.5 and M/S = 1-1.5 = 4

Component	Score	Scoring criteria
		P/S = 0.8-1.7 and M/S = 0.8-1.7 = 2
		Otherwise = 0

Note: RDA (Recommended dietary allowance), RNI (Recommended nutrient intake), AI (adequate intake)

A: red and processed meats/poultry meats/seafood/eggs; dairy products/beans; grains; fruits; vegetables.

B: red and processed meats, poultry meats, seafood, dairy products, beans, eggs.

C: carbohydrate: protein: fat.

D: PUFA (Poly-unsaturated fatty acid): MUFA (Mono-unsaturated fatty acid): SFA (saturated fatty acid).

Supplementary Table S3. Sex on dietary knowledge, attitude and practice scores.

Variable	Sex (M(IQR))		*P*
	Male (n = 271)	Female (n = 755)	
Dietary knowledge	83.3 (41.6)	83.3 (33.3)	<0.001
Dietary attitude	63.6 (13.6)	59.1 (13.6)	0.348
Dietary practice	71.7 (13.3)	71.5 (10.0)	0.412

Notes: M (median); IQR (interquartile range).

Supplementary Table S4. Student college year on dietary knowledge, attitude and practice scores.

Variable	College year (M(IQR))					*P*
	1st	2nd	3rd	4th	5th	
	(n = 228)	(n = 511)	(n = 100)	(n = 144)	(n = 44)	
Dietary knowledge	83.3 (41.7)	83.3 (33.3)	83.3 (33.3)	83.3 (25.0)	95.8 (16.7)	<0.001
Dietary attitude	59.1 (13.6)	63.6 (13.6)	61.4 (9.1)	59.1 (13.6)	63.6 (18.2)	0.119
Dietary practice	71.7 (13.3)	71.7 (13.3)	71.7 (11.3)	71.7 (9.6)	70 (12.9)	0.421

Notes: M (median); IQR (interquartile range).

Supplementary Table S5. Student college major on dietary knowledge, attitude and practice scores.

Variable	Major (M(IQR))			*P*
	Literature and art (n = 44)	Science and engineering (n = 78)	Medicine-related (n = 904)	
Dietary knowledge	66.7 (25.0)	83.3 (35.4)	83.3 (33.3)	<0.001
Dietary attitude	63.6 (13.6)	63.6 (22.7)	59.1 (13.6)	0.308
Dietary practice	70.8 (12.9)	71.7 (13.3)	71.7 (10.0)	0.124

Notes: M (median); IQR (interquartile range).

Supplementary Table S6. Different medicine-related majors on dietary knowledge, attitude and practice scores.

Variable	Medicine-related major (M(IQR))					*P*
	Clinical medicine (n=154)	Preventive medicine (n=425)	TCM (n=122)	Nursing (n=109)	Medical technology and information engineering （n=22）	
Dietary knowledge	83.3 (33.3)	83.3 (33.3)	100.0 (16.7)	91.7 (25.0)	75.0 (18.8)	<0.001
Dietary attitude	63.6 (18.2)	63.6 (13.6)	13.0 (13.6)	13.0 (13.6)	63.6 (13.6)	0.056
Dietary practice	73.3 (11.7)	71.7 (11.7)	71.7 (11.7)	71.7 (11.7)	70.0 (11.7)	0.202

Notes: M (median); IQR (interquartile range).

Supplementary Table S7. Dietary knowledge, attitude and practice scores of the participants.

Variable	(M (IQR)) or (mean ± SD)		*P*
	Intervention	Control	
Dietary knowledge (day 0)	100.0 (14.3)	100.0 (14.3)	0.672
Dietary attitude (day 0)	61.1 (11.1)	66.7 (11.1)	0.730
Dietary practice (day 0)	73.9 ± 6.5	75.6 ± 8.6	0.422
Dietary knowledge (day 3)	80.0 (13.3)	66.7 (13.3)	0.002
Dietary attitude (day 3)	66.7 (11.1)	61.1 (16.7)	0.058
Dietary practice (day 3)	74.0 ± 7.8	72.3 ± 9.0	0.367
Dietary knowledge (day 100)	66.7 (20.0)	60.0 (20.0)	0.023
Dietary attitude (day 100)	72.2 (16.7)	61.1 (5.5)	0.044
Dietary practice (day 100)	74.5 ± 8.0	70.8 ± 8.5	0.052

Notes: M (median); IQR (interquartile range); SD (standard deviation).

Supplementary Table S8. Intervention on participants' dietary quality scores.

Dietary quality		Difference (mean ± SD)	95% CI	*P*
Intervention group	day 0 vs day 3	3.0 ± 1.3	0.3 to 5.7	0.029
	day 3 vs day 100	-4.0 ± 1.0	2.0 to 6.0	<0.001
	day 0 vs day 100	-1.0 ± 1.4	-3.8 to 1.8	0.466
Control group	day 0 vs day 3	-0.1 ± 1.2	-2.4 to 2.5	0.965
	day 3 vs day 100	-1.4 ± 1.0	-0.6 to 3.4	0.157
	day 0 vs day 100	-1.4 ± 1.3	-4.1 to 1.2	0.276

Notes: SD (standard deviation); CI (confidence interval).

Supplementary Table S9. Dietary quality score comparison.

Visit	Group (mean ± SD)		*P*
	Intervention (n = 41)	Control (n = 41)	
day 0	50.6 ± 9.1	51.4 ± 9.4	0.654
day 3	53.5 ± 6.2	51.4 ± 7.9	0.158
day 100	49.5 ± 6.8	50.0 ± 9.0	0.798

Notes: SD (standard deviation).
